# Drought Responsive Putative Marker-Trait Association in Tall Fescue as Influenced by the Presence of a Novel Endophyte

**DOI:** 10.3389/fpls.2021.729797

**Published:** 2021-10-20

**Authors:** Shyamal K. Talukder, Md. Shofiqul Islam, Nick Krom, Junil Chang, Malay C. Saha

**Affiliations:** ^1^Grass Genomics, Noble Research Institute LLC, Ardmore, OK, United States; ^2^Texas A&M AgriLife Research Center, Beaumont, TX, United States; ^3^Scientific Computing, Noble Research Institute LLC, Ardmore, OK, United States

**Keywords:** tall fescue, drought, endophyte, marker trait association (MTA), stress tolerance

## Abstract

Tall fescue (*Festuca arundinacea* Schreb.) is one of the most important cool-season perennial obligatory outcrossing forage grasses in the United States. The production and persistence of tall fescue is significantly affected by drought in the south-central United States. Shoot-specific endophyte (*Epichloë coenophiala*)-infected tall fescue showed superior performance under both biotic and abiotic stress conditions. We performed a genome-wide association analysis using clonal pairs of novel endophyte AR584-positive (EP) and endophyte-free (EF) tall fescue populations consisting of 205 genotypes to identify marker-trait associations (MTAs) that contribute to drought tolerance. The experiment was performed through November 2014 to June 2018 in the field, and phenotypic data were taken on plant height, plant spread, plant vigor, and dry biomass weight under natural summer conditions of sporadic drought. Genotyping-by-sequencing of the population generated 3,597 high quality single nucleotide polymorphisms (SNPs) for further analysis. We identified 26 putative drought responsive MTAs (17 specific to EP, eight specific to EF, and one in both EP and EF populations) and nine of them (i.e., V.ep_10, S.ef_12, V.ep_27, HSV.ef_31, S.ep_30, SV.ef_32, V.ep_68, V.ef_56, and H.ef_57) were identified within 0.5 Mb region in the tall fescue genome (44.5–44.7, 75.3–75.8, 77.5–77.9 and 143.7–144.2 Mb). Using 26 MTAs, 11 tall fescue genotypes were selected for subsequent study to develop EP and EF drought tolerant tall fescue populations. Ten orthologous genes (six for EP and four for EF population) were identified in *Brachypodium* genome as potential candidates for drought tolerance in tall fescue, which were also earlier reported for their involvement in abiotic stress tolerance. The MTAs and candidate genes identified in this study will be useful for marker-assisted selection in improving drought tolerance of tall fescue as well opening avenue for further drought study in tall fescue.

## Introduction

Tall fescue, an outbreeding hexaploid (2n = 6x = 42) member of the Poaceae grass family, is one of the most economically important cool-season forage grass species, covering about 14 million hectares in the United States (Ball et al., 1991). It can adapt to a wide range of environments in the subtropical to temperate transition zone. It requires a minimum of 80–90 cm of annual rainfall and optimum growth temperatures of 20–25°C for high biomass production and longer persistence (Rogers and Locke, 2013). Thus, lack of precipitation and extreme heat during summer are major constraints for adaptation of tall fescue in the southern Great Plains of the United States. The extreme hot and severe droughts during summer months are common in south-central Oklahoma and Texas that cause complete stand loss of cool-season grasses. For example, in 2011, the south-central United States experienced historically exceptional heat and drought conditions, which caused $7.62 billion in agricultural losses and $3.23 billion in losses to the cattle sector in Texas^1^as well as $1.6 billion in agricultural losses in Oklahoma^2^.

Improving plant persistence under drought stress is therefore a key objective in tall fescue breeding programs for sustainable livestock production in drought-prone regions. Drought tolerance is a complex quantitative trait, which is governed by both genetic and environmental factors. Drought stresses during plant growth in the field are variable from time to time and affect gene expression of the drought tolerance related traits. The presence of an asymptomatic novel endophyte *Epichloë coenophiala* (Leuchtmann et al., 2014), which live in the intercellular space within leaves and pseudo-stems of tall fescue, helps the host plant to be tolerant toward biotic and abiotic stresses as well as conveys excellent persistence, improved vigor, and increased biomass productivity (Clay, 1990; Dinkins et al., 2017). In return, the host grasses provide photosynthates and nutrients to the endophytes (Schardl et al., 2004). Studies evaluating the effect of novel endophytes on tall fescue reported that endophyte-positive (EP) tall fescue showed greater tolerance to drought stress (Maclean et al., 1993; West et al., 1993; Buck et al., 1997; Bayat et al., 2009; Nagabhyru et al., 2013; Xu et al., 2017), enhanced resistance to pathogens under elevated CO_2_ (Chen et al., 2017), an increase in tiller number (Arachevaleta et al., 1989; Nagabhyru et al., 2013), and improved soil N (Arachevaleta et al., 1989) and P (Malinowski and Belesky, 1999) uptake compared to their endophyte-free (EF) counterpart. In addition to that physiological and phenotypic changes, such as altered root system architecture (Malinowski et al., 1999) and increased photosynthesis (Richardson et al., 1993), changes in metabolites (e.g., sugar, polyol, and amino acid concentrations) (Nagabhyru et al., 2013) were also observed with the presence of endophyte. Though previous studies examined the effect of endophyte on drought tolerance in tall fescue under controlled condition, but the quantitative trait loci responsible for drought tolerance in EP and EF tall fescue is unknown to date, to the best of our knowledge.

Recent advances in high-throughput sequencing technologies have accelerated genetic research by drastically reducing the cost of genome-wide marker discovery. This allowed the utilization of a genome-wide association study (GWAS) to dissect the genetic mechanism of agronomically important traits and to identify marker-trait associations (MTAs) to be used in breeding programs to develop improved cultivars. GWAS has been conducted in maize (Beló et al., 2008), sorghum (Tao et al., 2020), rice (Pantalião et al., 2016), perennial ryegrass (Jaškūnė et al., 2020, 2021), and tall fescue (Li et al., 2017) for the selection and improvement of desirable phenotypic traits.

In the present study, we evaluated clonal pairs of EP and EF tall fescue populations under natural summer conditions with sporadic drought to identify the genetic loci/SNPs that are associated with drought tolerance in tall fescue. Specifically, we aimed to (i) identify MTAs responsible for drought tolerance related traits in EP and EF tall fescue using GWAS, and (ii) identify orthologous genomic regions with annotation in the *Brachypodium* genome to identify candidate genes underlying the MTAs involving drought-tolerance-related traits in EP and EF tall fescue. The present study was conducted in natural field conditions and, to the best of our knowledge, this is the first GWAS conducted on clonal copies of EP and EF populations to understand the genetics and biology of the symbiotic relationship between tall fescue and endophytes that help the plants to survive under drought and heat stresses.

## Materials and Methods

### Plant Materials

The NFTD07 tall fescue population, consists of 203 progenies, was developed by crossing the drought-sensitive W947 and the drought-tolerant B348 genotypes. The W947 and B348 genotypes were selected from the PDF584 population, later released as cv. “Texoma MaxQ II” (Hopkins et al., 2011), which was a collection from southern Oklahoma, United States. The NFTD07 population contained a novel endophyte strain, AR584 (*E. coenophiala*) (Latch et al., 2000), that does not produce ergot alkaloid. Two sets of clonal ramets of the entire NFTD07 population were developed in the greenhouse of Noble Research Institute, LLC. One set was maintained as EP. In the other set, the inherent endophyte was removed using a hydroponic system as described in De Battista et al. (1990b) and the population was maintained as EF. Briefly, the EF tall fescue clones were developed using Tebucon 3.6F fungicide (Repar Corporation, Silver Spring, MD 20910) treatment (100 μL L^–1^ fertilizer solution) and after 1–2 weeks of fungicide treatment, EF clones were transferred into the soil tray. All the developed clonal pairs were confirmed for EP and EF using immunoblot (An et al., 1993) and PCR assays (Takach et al., 2012; Charlton et al., 2014). The confirmed EP and EF clonal ramets of each genotype were clonally propagated to make plants available for two treatments (EP and EF) with three biological replicates of each treatment, established in cones (175 mm × 51 mm) filled up with Metro-Mix 380^3^ and maintained for 3 weeks in the greenhouse before field planting.

### Field Evaluation

The field experiment was established at Noble’s Research Park located in Ardmore, Oklahoma (Latitude 34°11′33″N, Longitude 97°5′8″W, and 266 m above sea level) in November 2014. After adequate tillage and land leveling, clonal ramets of each of 205 EP and EF genotypes of the NFTD07 population, including their parents, were planted following a split plot arrangement in Randomized Complete Block Design with three replications, where endophyte status was considered as main plot and genotypes as subplot treatments. The distance between each plant in the field was one meter. The field was irrigated every other day using water guns for a week to ensure establishment of the ramets in the field. After this establishment period, plants were grown in rain-fed conditions. The field was properly managed for voluntary weeds, pests, and diseases. A soil test was conducted before transplanting and NPK fertilizer was incorporated based on the standard soil test index recommendations for Oklahoma (OSU Soil Test Interpretations | Oklahoma State University).

### Phenotyping and Weather Data Collection

**FIGURE 1:**
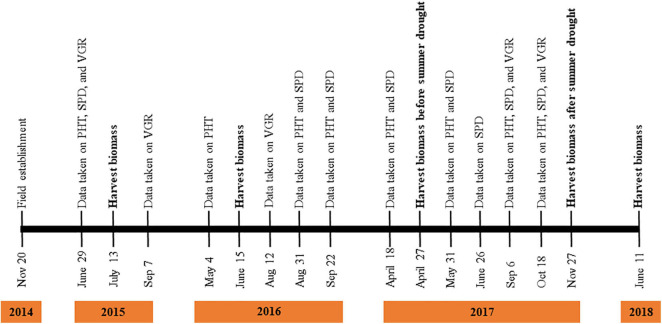
Time points of phenotypic data collection from field experiment established in November 2014 and ended in June 2018. PHT, SPD, and VGR indicates plant height, plant spread, and plant vigor, respectively.

Oklahoma receives minimal rainfall from June through August, and plants suffer sporadic to severe drought stress from July through September. Thus, the experiment was conducted under natural summer drought conditions, and phenotypic data related to drought stress were captured during August and September depending on the year of collection. The experiment was performed through November 2014 to June 2018 in the field (Figure 1), and data on plant height (PHT), plant spread (SPD), and plant vigor (VGR) were collected after a no-rain window of at least 3 weeks in August and September across the growing season whenever possible (daily rainfall data for the study period can be obtained from the Ardmore Mesonet website *Mesonet*). The VGR was scored by two people independently on a 0–10 scale, where 0, dead; 1–3, very stressed; 4–5, stressed; 6–7, leaf yellowing; 8–9, leaf curling; and 10, plant exceptionally vigorous and no sign of stress. The PHT was measured in centimeters from the base of the tiller to the tip of the tallest leaf blade. The SPD was measured in centimeters in the widest part of the plant cover. Plants were harvested for fresh biomass using a lightweight gas hedge trimmer with a long blade [HL 94 (145°), STIHL, United States] before and after summer drought. Fresh biomass of each genotype was oven-dried at 55°C for 5–7 days to obtain dry biomass weight (DWT). All the phenotyping data of the NFTD07 population were collected from the EP and EF ramets separately.

The mean monthly temperatures and cumulative monthly precipitation were collected from the Mesonet weather station (Station Number 126) located in Ardmore, Oklahoma, United States, which is adjacent to the experimental site^4^ from field establishment year (2014) to experiment end year (2018). In addition, 14-year (2005–2018) mean data on temperature and total precipitation at the experimental site were also collected to compare growing season weather data with long-term average.

### Statistical Analysis

The best linear unbiased prediction (BLUP) of each genotype for each trait in each growing season of the population was calculated using the *lmer* function in R package *lme4* (Bates et al., 2015). To identify significant differences between the mean of each trait in the EP and EF populations, Student’s *t*-tests were carried out using Excel. The standard deviation (SD) of a population mean for each trait was calculated using R software.

As phenotypic data of EP and EF tall fescue were collected in multiple years (Figure 1), of which 2 year data of PHT and SPD (September 2016 and September 2017) and VGR (August 2016 and September 2017) collected after or within drought effect were used to perform analysis of variance (ANOVA) using PROC GLM in SAS system 9.3 (SAS, 1999). For each trait, genotype, genotype × year, genotype × endophyte × year interaction and errors were assumed as a random effects. From ANOVA, phenotypic variance was calculated. Pearson’s correlation of traits during summer months was carried out using ggcorrplot function (Cai, 2019) in RStudio.

### DNA Extraction

Young leaf tissue from the 205 tall fescue genotypes was collected in a brown paper envelop, freeze-dried, and ground to a fine powder using a Mixer Mill Type MM 300 (Retsch, Hann, Germany). Genomic DNA was extracted using a DNeasy^®^ Plant Mini-prep DNA Extraction Kit (QIAGEN Inc., Valencia, CA, United States) according to the manufacturer instructions. DNA concentrations were quantified using a NanoDrop ND 1000 Spectrophotometer (NanoDrop Technologies, Wilmington, DE, United States).

### Genotyping-by-Sequencing Libraries, Sequencing, and Single Nucleotide Polymorphisms Calling

Genotyping-by-sequencing (GBS) was performed using Illumina HiSeq2000 paired-end sequencing (2 bp × 100 bp) platform following the method described in Elshire et al. (2011) in the Institute for Genomic Diversity, Cornell University, Ithaca, NY, United States, in 2016. Briefly, 200 ng genomic DNA of each sample at the rate of 20 ng μL^–1^ were digested with *Ape*KI restriction endonuclease enzyme (New England BioLabs, Inc., Hitchin, United Kingdom). Digested samples were ligated with a barcoded adapter using T4 ligase (New England BioLabs). After ligation, 96 samples were pooled to a single tube and PCR amplified. The 96-plex library per flow cell channel was used for sequencing.

The GBS reads were quality-filtered to a minimum read length of 82 bp after adapter and barcode trimming and minimum base quality of 20. SNPs were identified using the default parameters of Stacks (v1.37) *de novo* pipeline (Catchen et al., 2013). The identified SNPs with missing data more than 30%, read depth less than 10, and minor allele frequency (MAF) less than 0.05 were removed from the analysis using VCFtools (v0.1.13) (Danecek et al., 2011). The missing data of the GBS markers in the population were imputed using LinkImpute software package based on LD-kNNi, a method for *k*-nearest neighbor genotype imputation designed for unordered markers (Money et al., 2015).

### Population Genetic and Genome-Wide Association Analysis

We calculated population structure (Q) using principal component (PC) analysis and relationships among genotypes through a kinship (K) matrix using default parameters of TASSEL 5.0^5^ software. To identify the optimum number of PCs to use in GWAS analysis, a scree plot was constructed by plotting the percentage of variances explained by the first 10 PCs to the number of PCs. The optimum number of PCs was selected where the “elbow” point occurred. The analysis of K between genotypes was performed using 3,597 SNP markers identified in the tall fescue genome.

The GWAS analysis was conducted using TASSEL 5.0 software (Bradbury et al., 2007) by implementing Q + K method as a mixed linear model (MLM) model. For the Q method, the first three PCs (PC_1_, PC_2_, and PC_3_) that were determined from scree plot constructed from PCs were included in the model as fixed-effect covariates (Zhao et al., 2007) to adjust population stratification. In the K method, the relationship between genotypes was calculated and included in the model as random-effect covariate. The BLUP values of the replicated field experiments were used for the GWAS. The Manhattan and quantile-quantile (Q-Q) plots were created for each trait from GWAS results using TASSEL 5.0. The *p*-values were extracted from the TASSEL’s output and a suggestive genome-wide threshold level of *p*-value < 0.001 or –log_10_ (*p*-value) ≥ 3.00 was considered for significance MTAs considering the deviation of the observed values from the expected values in the Q-Q plots of the present study. Further, each significant MTA was subjected to Bonferroni correction threshold –log_10_ (*p*-value) ≥ 4.9 at significance level 0.05 for eliminating the false positives. The proportion of the explained phenotypic variation by each marker was estimated by the relevant *R*^2^. Among them, the MTAs identified only during dry and extreme hot conditions (July through September) and absent during other times of the year were considered drought-responsive MTAs with and/or without the presence of endophyte in the NFTD07 population.

### Selection of Drought Tolerant Tall Fescue Genotypes

The drought tolerant alleles from of the 26 MTAs were manually checked in D07 population in order to identify drought tolerant tall fescue genotypes for further study to develop EP and EF drought tolerant populations.

### *In silico* Validation and Identification of Tall Fescue Variants Within *Brachypodium* Genome

One consensus sequence of each stack length (82 bp) obtained from the Stacks program (Catchen et al., 2013) was selected for each SNP locus. The consensus sequences were then aligned against *Brachypodium distachyon* genome (version 3.0)^6^ using NCBI BLASTn version 2.2.25. Sequence only with single alignment of the highest match with *E*-value ≤ 0.001 was selected as the respective SNP’s position in the *Brachypodium* genome. To identify orthologous gene(s) of the MTAs responsible for drought tolerance, SNP position was determined manually based on the genomic coordinates against *Brachypodium* genome annotation in GFF format downloaded from the NCBI GenBank^7^. When the SNP position was found in the genic region, the gene was identified as the orthologous gene of the respective MTAs. The orthologous gene(s) could be the candidate gene(s) responsible for drought-tolerance-related traits in EP and EF populations.

## Results

### Phenotypic Trait Variation in the Endophyte-Positive and Endophyte-Free Clonal Pairs

This experiment was conducted under natural drought conditions, but the rainfall data suggested that we had a fairly dry summer (June through September) with average summer monthly rainfall of 14.48, 7.56 and 8.19 cm in 2015, 2016, and 2017, respectively, with at least a mostly dry month (August 2015, July 2016, and September 2017) in each year (Supplementary Table 1). The mean temperatures in at least 1 month (July 2015, 28.67°C; July 2016, 29.22°C; and July 2017, 28.28°C) and the sporadic drought during summer in each year hampered normal growth and developments of the plants.

A wide phenotypic variability was found for all the measured traits, i.e., PHT, SPD, VGR, and DWT. In most cases, data showed normal distributions (Supplementary Figure 1) and the EP plants showed higher values than the EF plants except DWT Jul15 and DWT Nov17 (Table 1). The average PHTs of EP population were significantly higher (*p* < 0.05) than the EF population at every time the data were collected. Plants of EP population were 1.14–11.74% taller than those of EF population across years. The height advantage of EP plant was higher (10.67% in Aug 2016 and 11.74% in Sep 2016) when they grew through summer drought. The advantage was lower (2.70% in May 2016 and 1.14% in May 2017) when they grew under abundant rainfall and optimum air temperature (Table 1 and Supplementary Table 1).

**TABLE 1:** Phenotypic variation of plant height (PHT), plant spread (SPD), plant vigor (VGR), and dry biomass weight (DWT) between clonal pair of EP and EF tall fescue population under natural summer with sporadic drought condition in the growing season, 2015–2018.

**Traits**	**Data collection^‡^**	**EP**			**EF**			**EP > EF (%)**
		**Range**	**Mean**	**SD**	**Range**	**Mean**	**SD**	
PHT	June 15	87.20–106.80	98.69*	3.39	76.69–106.28	95.75	4.64	2.98
	May 16	101.95–143.74	128.85*	6.39	87.06–140.26	125.37	6.79	2.70
	August 16	29.80–49.85	38.62*	3.20	27.88–40.46	34.50	2.23	10.67
	September 16	30.79–53.88	40.47*	3.79	26.10–46.86	35.72	3.19	11.74
	April 17	42.61–56.02	48.73*	2.28	38.04–52.65	44.46	2.58	8.76
	May 17	33.33–45.88	38.72*	2.35	37.47–44.20	38.28	0.61	1.14
	September 17	35.62–41.32	39.97*	0.97	35.09–39.97	38.10	0.80	4.68
	October 17	35.62–41.46	39.57*	0.85	34.46–40.15	38.16	0.95	3.56
SPD	June 15	19.07–23.31	21.20*	0.79	17.33–23.22	20.73	0.95	2.22
	August 16	17.01–31.97	27.54*	2.19	19.04–29.09	24.81	1.91	9.91
	September 16	21.50–34.96	29.05*	2.25	15.48–31.01	25.28	2.57	12.98
	April 17	33.83–48.82	43.43*	1.89	34.45–44.59	40.08	2.01	7.71
	May 17	20.37–32.55	28.66*	1.53	22.95–29.98	26.95	1.00	5.97
	June 17	23.79–30.96	28.52*	0.96	22.90–29.44	27.16	1.24	4.77
	September 17	31.73–33.53	32.86*	0.29	26.71–32.61	30.71	0.86	6.54
	October 17	33.16–34.82	34.34*	0.21	30.48–34.45	33.41	0.65	2.71
VGR	June 15	7.19–8.29	7.78*	0.20	7.11–8.23	7.73	0.18	0.64
	September 15	4.25–6.11	5.19*	0.38	3.10–6.70	5.05	0.58	2.70
	August 16	5.07–8.02	6.82*	0.46	4.41–7.03	6.11	0.53	10.41
	September 17	5.60–6.97	6.41*	0.27	4.40–6.38	5.49	0.44	14.35
	October 17	5.20–6.86	6.15*	0.29	4.16–6.26	5.32	0.44	13.50
DWT	July 15	63.80–119.84	92.65	10.37	80.18–132.12	97.74*	8.50	−5.49
	June 16	232.08–333.27	278.05^*NS*^	17.15	215.08–330.59	277.19	21.10	0.31
	April 17	163.91–173.60	168.43*	1.84	154.38–184.61	167.59	5.30	0.50
	November 17	76.17–97.23	83.30	3.63	58.19–126.72	83.78^*NS*^	12.00	−0.58
	June 18	74.17–82.55	77.16*	1.65	59.08–101.46	71.94	5.83	6.77

*^‡^Month and year of the growing seasons when data were collected.*

*EP, endophyte-positive population; EF, endophyte-free population.*

*Indicate significant differences (Student’s *t-Test*, *p*-value ≤ 0.05) between the mean of EP and EF tall fescue populations.

*SD, standard deviation.*

*NS, non-significant.*

The average SPDs of EP population were significantly (*p* < 0.05) higher than the EF population throughout the experiment. The EP population had SPD advantage over EF population, ranging between 2.22–12.98%. Similar to PHT, the SPD advantage of EP plants was higher (9.91% in August 2016 and 12.98% in September 2016) when they grew under drought stress. The advantage was lower (2.22% in June 2015 and 2.71% in Oct 2017) when they grew under optimum growing condition (Table 1 and Supplementary Table 1). All VGR traits showed significant differences (*p* < 0.05) between EP and EF population, where EP population had higher VGR advantage than EF population varied from 0.64 to 14.35%. Similar to PHT and SPD traits, we observed the highest VGR advantage of EP population (14.35%) in September 2017 and the lowest (0.64%) in June 2015 (Table 1).

Plant growth was severely affected and did not grow much during August 2015 (total rainfall 0.53 cm) and July 2016 (total rainfall 1.14 cm). To prevent stand loss, plant biomass was not harvested after summer months in those years. The average DWT was significantly different (*p* < 0.05) between EP and EF population except June 2016 and November 2017 (Table 1). In 2017, plant biomass harvesting was done in April and in November. After drought effect, the mean DWT of November 2017 harvest was 83.30 and 83.78 g in EP and EF populations, respectively. The EP population showed significant advantage in DWT of June 2018 harvest (6.77%) over EF population for dry biomass production (Table 1). Analysis of variance for PHT, SPD and VGR recorded under sporadic drought condition in September for years 2016 and 2017 of EP and EF tall fescue showed significant (*p* < 0.05) effect in all components except genotype × endophyte × year interaction (Table 2).

**TABLE 2:** Analysis of variance with mean square for drought traits of EP and EF tall fescue genotypes grown under rain fed condition in Ardmore, Oklahoma, United States in 2016 and 2017.

**Traits**	**Abbrev.**	**Genotype (G)**	**Endophyte (End)**	**Year**	**G × Year**	**G × End**	**G × End × Year**	**Error**
Plant height (cm)	PHT	172.74***	6,845.12***	538.39**	87.6***	70.7*	56.12^*ns*^	56.8***
Plant spread (cm)	SPD	97.56***	5,408***	12,139.56***	39.41*	45.65***	25.38^*ns*^	31.65***
Plant vigor (score)^‡^	VGR	8.3***	426.67***	175.4***	3.58^*ns*^	4.39**	2.41^*ns*^	3.1***

*, **, and *** significant at 0.05, 0.01, and 0.001 probability level, respectively; ns, non-significant.

*^‡^VGR data included from August 2016 and September 2017, as both collection time were in the summer month.*

### Correlation of Phenotypic Traits

**FIGURE 2:**
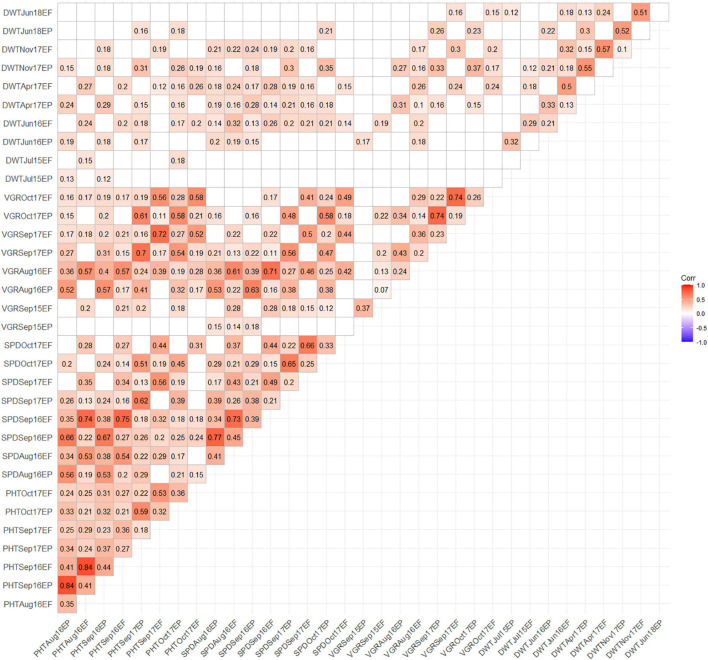
Correlation matrix showing association among drought related traits obtained using phenotypic data collected from 205 EP and EF tall fescue genotypes. Insignificant (*p*-value > 0.05) correlation coefficient values are presented with a blank block.

The Pearson’s correlation coefficient revealed association between growth and yield traits amongst the EP and EF populations under drought condition (Figure 2). In all cases, phenotypic traits showed significant (*p* < 0.05) positive and insignificant correlation within EP and EF population between traits. No negative correlation between traits was detected in any of the years. In EP population, PHTSep17 showed higher correlation with VGRSep17 (*r* = 0.7) and low with DWTNov17 (*r* = 0.31). The SPDSep17 exhibited higher correlation with VGRSep17 (*r* = 0.58) and low with DWTNov17 (*r* = 0.3). Likewise, in the EF population, PHTAug16 showed higher correlation with SPDSep16 (*r* = 0.74) and PHTSep17 exhibited higher correlation with VGRSep17 (*r* = 0.72). The VGRSep17 revealed higher correlation with VGROct17 (*r* = 0.74) and lower with DWTNov17 (*r* = 0.3) (Figure 2).

### Single Nucleotide Polymorphisms Discovery and *in silico* Validation

From the GBS sequence data, a total of 2.5 million SNP markers were identified using the Stacks pipeline. After stringent filtering (read depth ≥ 10 and missing value ≤ 30), 11,312 SNPs retained. Among them, 3,597 SNPs with MAF ≥ 0.05 were considered for subsequent downstream analysis (Supplementary Table 2). Among the 3,597 tall fescue SNP markers, 1,355 SNPs were uniquely mapped on the five chromosomes of the *Brachypodium* genome. Among them, 403, 309, 305, 190, 148 SNPs were distributed in the *Brachypodium* chromosomes 1, 2, 3, 4, and 5, respectively. In general the tall fescue SNPs were well distributed throughout the *Brachypodium* chromosomes with less frequency in centromeric regions (Bd1: 37.57–38.30, Bd2: 28.66–29.46, Bd3: 25.22–25.49, Bd4: 20.67–20.95, and Bd5: 7.67–8.50 Mb) (Li et al., 2018) and high frequency in telomeric regions (Supplementary Figure 2).

### Genome-Wide Association Mapping for Drought Tolerance Related Traits

**FIGURE 3:**
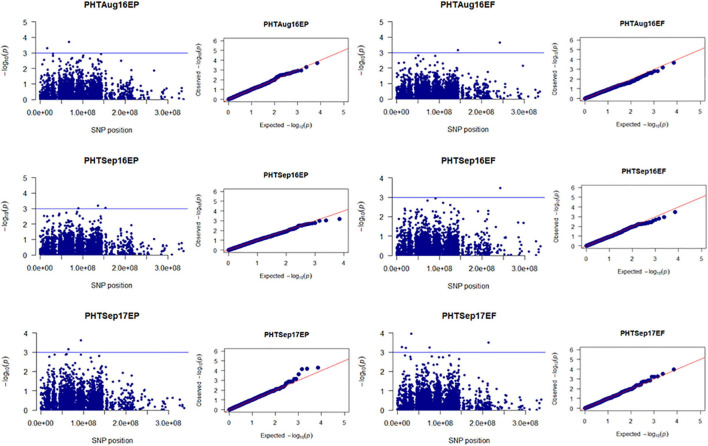
Manhattan and Q-Q plots showed drought-responsive MTAs on plant height. X-axis represents SNP position in the tall fescue *de novo* genome assembly performed by Stacks software, and Y-axis represents –log_10_ (*p*) values of the marker-trait associations. The black line represents the suggestive significant threshold level of –log_10_ (*p*) value = 3.0.

**FIGURE 4:**
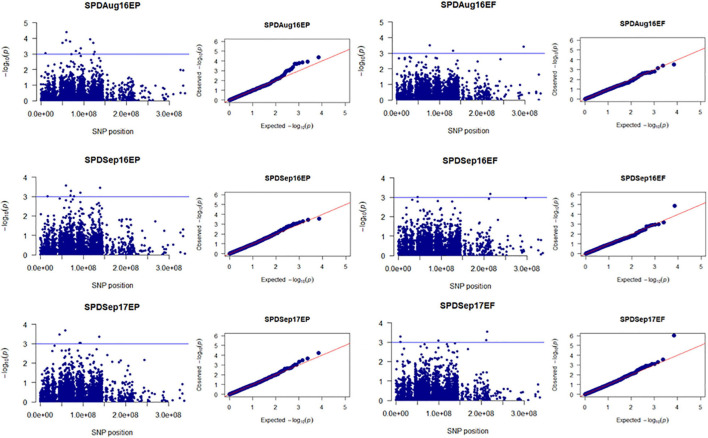
Manhattan and Q-Q plots showed drought-responsive MTAs on plant spread. X-axis represents SNP position in the tall fescue *de novo* genome assembly performed by Stacks software, and Y-axis represents –log_10_ (*p*) values of the marker-trait associations. The black line represents the suggestive significant threshold level of –log_10_ (*p*) value = 3.0.

**FIGURE 5:**
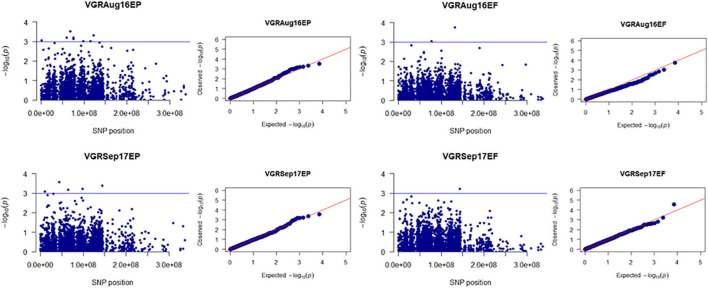
Manhattan and Q-Q plots showed drought-responsive MTAs on plant vigor. X-axis represents SNP position in the tall fescue *de novo* genome assembly performed by Stacks software, and Y-axis represents –log_10_ (*p*) values of the marker-trait associations. The black line represents the suggestive significant threshold level of –log_10_ (*p*) value = 3.0.

**FIGURE 6:**
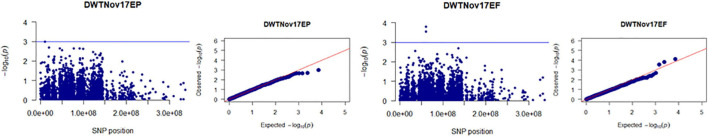
Manhattan and Q-Q plots showed drought-responsive MTAs on dry biomass weight. X-axis represents SNP position in the tall fescue *de novo* genome assembly performed by Stacks software, and Y-axis represents –log_10_ (*p*) values of the marker-trait associations. The black line represents the suggestive significant threshold level of –log_10_ (*p*) value = 3.0.

Population structure (Q) of 205 tall fescue genotypes was analyzed using 3,597 SNPs through PC analysis implemented in TASSEL software. The kinship analysis was sufficient for controlling the confounding effects of population structure in GWAS (Zhao et al., 2007; Abdel-Ghani et al., 2019). Variance explained by PC analysis were 2.7% for PC1, 1.8% for PC2, and 1.4% for PC3 (Supplementary Figure 3). The kinship (K) analysis identified two groups of plant materials of this study. Group one (G1) and group two (G2) contained 115 and 90 genotypes, respectively (Supplementary Figure 4). Using the MLM model (Q + K) in TASSEL, a total of 147 MTAs [having *p*-value less than 0.001 or suggestive threshold level of -log_10_ (*p*-value) ≥ 3.0] were identified for all four traits (PHT, SPD, VGR, and DWT) in 16 time points data across 4 years in both EP and EF populations (Figure 1 and Supplementary Table 3). Among which, 71 (48.3%) and 61 (41.5%) MTAs were specific to the EP and EF populations, respectively; and 15 (10.2%) MTAs were common in both populations. The explained phenotypic variation of the significant 147 MTAs ranged between 5–33%, ranged in EP and EF population were 5–12% and 6–33%, respectively. Based on MTAs only affecting traits under drought during summer months, 17 MTAs related to PHT, SPD, and VGR in EP; eight MTAs related to PHT, SPD, VGR, and DWT in EF; and one MTA for SPD in both populations were considered to be putative drought responsive (Figures 3–6 and Supplementary Table 3). The explained phenotypic variation of the drought responsive 26 MTAs ranged between 6–13% (Supplementary Table 3). The highest explained phenotypic variation (28%) of drought responsive MTA (HSV.ef_31) was found for three traits such as PHT (6%), SPD (13%), and VGR (9%).

Out of 147 MTAs, only 12 MTAs passed Bonferroni correction threshold (Supplementary Table 3) of which three (H.ep_2, SV.ep_26, and S.ep_71) belonged to EP population, three (H.ef_19, H.ef_29 and HSV.ef_31) belonged to EF population, and the remaining six (HS_2#, HSV_3#, HSV_6#, HSV_10#, H_11#, and HSV_12#) belonged to both EP and EF population. Out of these 12 MTAs, only one (HSV.ef_31) that controlled SPD in the EF population was detected under drought condition. No MTA detected under drought condition in the EP population, passed Bonferroni correction threshold. This indicates that the Bonferroni correction test is too stringent in this field study.

**FIGURE 7:**
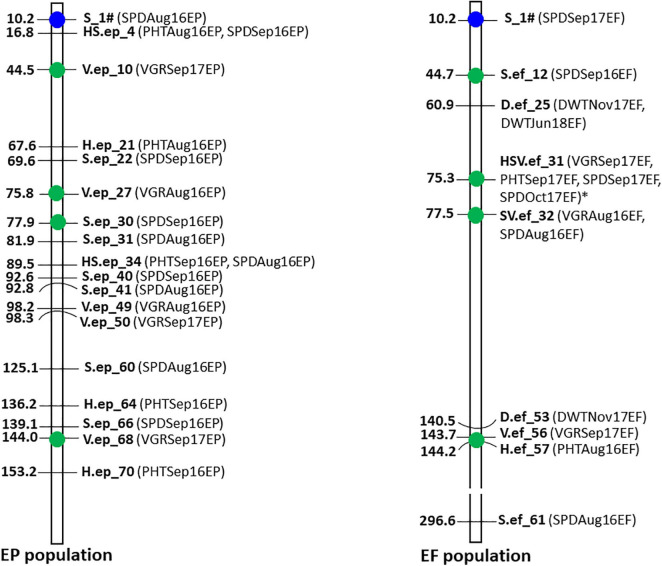
Map of 26 significant markers associated with drought tolerance traits under natural summer drought in the EP and EF tall fescue populations. Marker positions were shown in Mb on the left side and trait names were shown on the right side of the bar. Marker with blue circle was common in both populations. Green-colored circle indicates MTAs within 0.5 Mb region between EP and EF populations. All other MTAs were specific to EP or EF population. MTA with asterisk mark passed the Bonferroni correction test at significance level 0.05.

When more than one MTA were detected within 0.5 MB region in the contig assembly, we defined the regions as MTA hotspots. There were four hotspots (Figure 7) identified for drought responsive MTAs consisting total of nine MTAs in those regions as 44.5–44.7 Mb (V.ep_10 and S.ef_12); 75.3–75.8 Mb (V.ep_27 and HSV.ef_31); 77.5–77.9 Mb (S.ep_30 and SV.ef_32); and 143.7–144.2 Mb (V.ep_68, V.ef_56, and H.ef_57).

### Selection of Drought Tolerant Tall Fescue Genotypes

In this study, based on the presence of highest number of drought related MTAs, nine genotypes (D07001, D07029, D07090, D07108, D07146, D07155, D07181, D07187, and D07225) were selected from both the EP and EF populations and other two (D07053 and D07242) were selected from only EP population. These 11 genotypes were subsequently maintaining in the Noble Research Institute, Ardmore, Oklahoma greenhouse in order to develop EP and EF drought tolerant populations for further studies.

### Prediction of Candidate Genes for Drought Tolerance Traits

To identify candidate genes associated with drought tolerance traits, 26 drought responsive MTAs were searched in the *Brachypodium* genome. Based on the genomic coordinates of the consensus sequences, we were able to identify orthologous genes for 10 MTAs from the *Brachypodium* genome, of which, six were specific to EP population and four genes linked to EF population (Table 3). In the EP population, the uncharacterized protein *LOC104583950* located at 44.5 Mb region was a candidate gene for VGR in September 2017, the acetylajmalan esterase (*BRADI_4g18187*) located at 69.6 Mb region for SPD in September 2016, dynamin-related protein 4C (*BRADI_1g67517*) located at 89.5 Mb region for PHT in September 2016 and SPD in August 2016, SAC3 family protein C (*BRADI_1g62340*) located at 98.2 Mb region for VGR in August 2016, protein altered xyloglucan 4-like (*BRADI_1g44920*) located at 125.1 Mb region for SPD in August 2016, and aspartyl protease AED3 (*BRADI_1g19070*) located at 144.0 Mb region for VGR in September 2017. In the EF population, the uncharacterized glycosylphosphatidylinositol (GPI)-anchored protein (*BRADI_1g42478*) located at 77.5 Mb region, ortholog of *Arabidopsis At4g28100*, was a candidate gene for both SPD and VGR in August 2016, the calmodulin-like protein 5 (*BRADI_3g58086*) located at 140.5 Mb region for DWT in November 2017, the phospholipase D zeta 1 (*BRADI_2g11807*) located at 143.7 Mb region for VGR in September 2017, and the putative RING-H2 finger protein ATL12 (*BRADI_2g14170*) located at 296.6 Mb region for SPD in August 2016 (Table 3).

**TABLE 3:** Candidate genes associated with the selected MTAs contributed to plant height, plant spread, plant vigor, and dry biomass weight obtained at natural summer drought condition using GWAS.

	Traits with time points and endophyte status*	**SNP markers**	**SNP alleles**	**Based on *Brachypodium* genome information**	
				**Orthologous gene^ϕ^**	**Description**
**Both EP and EF populations**					
S_1#	SPDAug16EP, SPDSep17EF	SNP_10249894	C/G	NF	–
**EP population**					
HS.ep_4	PHTAug16EP	SNP_16824944	C/G	NF	–
	SPDSep16EP				
V.ep_10	VGRSep17EP	SNP_44518876	T/A	*LOC104583950*	Uncharacterized protein LOC104583950
H.ep_21	PHTAug16EP	SNP_67685857	G/A	NF	–
S.ep_22	SPDSep16EP	SNP_69669107	C/G	*BRADI_4g18187*	Acetylajmalan esterase
V.ep_27	VGRAug16EP	SNP_75856836	C/T	NF	–
S.ep_30	SPDSep16EP	SNP_77992676	G/A	NF	–
S.ep_31	SPDAug16EP	SNP_81925080	G/A	NF	–
HS.ep_34	PHTSep16EP	SNP_89594481	G/A	*BRADI_1g67517*	Dynamin-related protein 4C
	SPDAug16EP				
S.ep_40	SPDSep16EP	SNP_92621494	C/T	NF	–
S.ep_41	SPDAug16EP	SNP_92829771	C/T	NF	–
V.ep_49	VGRAug16EP	SNP_98293586	C/T	*BRADI_1g62340*	SAC3 family protein C
V.ep_50	VGRSep17EP	SNP_98358487	G/A	NF	–
S.ep_60	SPDAug16EP	SNP_125100400	G/C	*BRADI_1g44920*	Protein altered xyloglucan 4-like
H.ep_64	PHTSep16EP	SNP_136209828	C/T	NF	–
S.ep_66	SPDSep16EP	SNP_139134835	A/T	NF	–
V.ep_68	VGRSep17EP	SNP_144067143	C/A	*BRADI_1g19070*	Aspartyl protease AED3
H.ep_70	PHTSep16EP	SNP_153274658	C/T	NF	–
**EF population**					
S.ef_12	SPDSep16EF	SNP_44725770	C/G	NF	–
D.ef_25	DWTNov17EF	SNP_60994487	C/G	NF	–
		SNP_60994497	C/A	NF	–
	DWTJun18EF	SNP_60994487	C/G	NF	–
		SNP_60994497	C/A	NF	–
HSV.ef_31	PHTSep17EF	SNP_75375405	A/G	NF	–
	SPDSep17EF				
	VGRSep17EF				
	SPDOct17EF				
SV.ef_32	SPDAug16EF, VGRAug16EF	SNP_77518826	C/A	*BRADI_1g42478*	Uncharacterized GPI-anchored protein *At4g28100*
D.ef_53	DWTNov17EF	SNP_140530490	G/A	*BRADI_3g58086*	Calmodulin-like protein 5
V.ef_56	VGRSep17EF	SNP_143720205	C/T	*BRADI_2g11807*	Phospholipase D zeta 1
H.ef_57	PHTAug16EF	SNP_144265489	T/C	NF	–
S.ef_61	SPDAug16EF	SNP_296672096	C/T	*BRADI_2g14170*	Putative RING-H2 finger protein ATL12

*The first three letters refer to trait name, the second three letters and two digit numbers indicate month and year of the growing seasons when data were collected; EP, endophyte-positive and EF, endophyte-free population.

*^ϕ^NF, SNP does not found in the *Brachypodium* genome; LOC104583950, alias gene name absent in the *Brachypodium* gene list.*

## Discussion

### Evaluation of Endophyte-Positive and Endophyte-Free Clonal Pairs Under Summer Drought Condition

Being a cool-season perennial grass species, drought tolerance in tall fescue is important for stand persistence during hot and dry summer months (July through September) in the southern Great Plains of the United States. The ability of regrowth of a tall fescue plant following a severe drought event depends on its drought tolerance or avoidance capability in the field. As a perennial crop, it should be productive and persist at least for five to 10 years once planted (Saha, 2018). However, 20–40% stand losses have been recorded every year due to hot and dry summers in the region (Saha, 2018). Previous research demonstrated that the EP tall fescue show better tolerance to drought compared to their EF counterparts, assessed by morphological traits such as number of tiller, leaf rolling, regrowth after harvest with abundant watering of drought-stressed plants, and plant spread (Arachevaleta et al., 1989; White et al., 1992). In this study, four agronomic traits were used to assess the survival ability of the clonal pairs of EP and EF tall fescue populations during hot and harsh summer season under natural summer drought condition.

Aligning with previous findings (Arachevaleta et al., 1989; White et al., 1992), we found significant positive influence of endophyte in tall fescue for PHT, SPD, and VGR under natural summer drought. It was interesting to observe that the endophyte had the largest influence in summer months, when plants went through summer drought condition. Given example, we observed highest advantage for PHT and SPD in August and September 2016 and for VGR in August 2016, September 2017, and October 2017 in EP population than EF population (Table 1 and Supplementary Table 1). De Battista et al. (1990a) reported that the fungal endophyte *E. coenophiala* produced growth hormone auxin, Indole Acetic Acid (IAA), which helps in cell elongation and cell division resulting tall and spreading type morphology observed in EP than EF tall fescue. West et al. (1993) also reported that endophyte contributes to make tall fescue stable through tiller and whole plant survival during drought stress. In contrast, we observed that endophyte had the smallest influence before summer months (May through June) and after summer months (October). It means plants may not need the endophyte support when they grow in optimum growing conditions (Table 1 and Supplementary Table 1).

In the case of DWT, we observed significant differences but mixed responses of endophyte for drought tolerance and/or avoidance. Both positive and negative influences of the endophyte might be due to the conjugative response of multiple factors. Despite the increment of PHT, SPD, and VGR, endophyte response for DWT in July 2015 and November 2017 was negative for DWT in the EP population. The following reasons may have influenced our results: (i) we were not able to harvest at the appropriate time, right after summer drought, to capture the endophyte effect due to adverse weather and field conditions and (ii) it was not possible to harvest all plants mechanically at the same height from the base, thus the above-ground biomass yield may not have been accurately captured. In short, the EP plants growing under summer drought tended to produce young tillers, look greener, become taller and stay softer. On the other hand, EF plants with less tolerance tended to grow faster before drought and become dry and stunted with stronger culms during summer; thus, they may contribute higher dry biomass during harvest. Arachevaleta et al. (1989) reported that common wild-type EP clones produced slightly more herbage than EF clones of Kentucky-31 tall fescue under normal growth condition, but they produced maximum 50% higher shoot biomass than their EF clones in the presence of higher available N. In addition, Belesky and Fedders (1995) found 15 and 11% increases in shoot and root biomass production, respectively, in wild-type EP clones relative to EF clones. In contrast, Guo et al. (2015) reported that shoot biomass weight were similar between clonal pairs of novel EP and EF tall fescue cv. PDF under controlled conditions. They concluded that biomass production with the presence of the novel endophyte are genotype-specific. Our results on DWT were in line with the finding of Guo et al. (2015), as similar genetic background plant materials were used in both studies. The accumulating research evidence suggests that mutual benefit between novel endophyte and tall fescue is conditional on biomass production, dependent on the genotype of host plant (Belesky and Fedders, 1996) as well as the N availability in soil (Saikkonen et al., 2006). Subsequently, Rasmussen et al. (2012) proposed that novel endophyte degrades host plant cell wall carbohydrates to achieve carbon as part of their symbiotic relationship. Our data on PHT, SPD, and VGR suggest that novel endophyte-positive clones were able to produce higher biomass than endophyte-free tall fescue clones, though we did not identify any MTA for DWT in EP population in this study.

### Single Nucleotide Polymorphisms Calling Using Stacks

Tall fescue exhibits disomic inheritance (Lewis et al., 1980; Sleper, 1985; Xu et al., 1995) and behaves like a diploid species. Stacks was thus used for SNP calling in tall fescue genome without any issues.

## Genome-Wide Association Analyses

In this study, we performed GWAS analysis in a tall fescue population. Because tall fescue is an obligate outbreeding forage grass, plants grown from different seeds are necessarily unique genotypes with sufficient genetic variability. We used 3,597 GBS-SNP markers with phenotypic data to identify MTAs for each trait using GWAS. As tall fescue is a perennial grass and the field condition of the growing seasons were continuously variable, GWAS analysis was performed separately in each growing season to capture the stable genetic effects on traits under target environment. Despite the fact that both EP and EF populations have the same genetic background, only 15 MTAs were found common in both populations for all the measured traits. This might be due to the interaction effect of endophyte vs. genotype, which was carried over and accumulated throughout the life cycle of tall fescue. Probably due to the same reason, the number of drought responsive MTAs in the EP population was almost double that of the EF population, and only one drought responsive MTA was common in both populations. Plant phenotyping was performed in sporadic drought conditions, which was highly variable during trait measurement and could be a major cause for observing fewer co-localized MTA in the study. Analyzing the drought responsive hotspot regions [44.5–44.7 Mb (V.ep_10 and S.ef_12); 75.3–75.8 Mb (V.ep_27 and HSV.ef_31); 77.5–77.9 Mb (S.ep_30 and SV.ef_32); and 143.7–144.2 Mb (V.ep_68, V.ef_56, and H.ef_57)], it was clear that MTAs associated with plant spread, plant vigor, and plant height were closely co-localized for data collected in different times and years, which was meaningful as those are highly correlated traits that reflected plant growth under summer drought.

## Identification of Orthologous Gene(s) Responsible for Drought Tolerance

The occurrence of MTAs for PHT, SPD, VGR, and DWT in the tall fescue genome suggest that we identified genomic regions containing gene(s) responsible for plant survival under unfavorable growth condition. Therefore, identification of candidate gene(s) using markers associated with traits may lead us to understand the genetic mechanism of drought tolerance in EP and EF tall fescue populations. As tall fescue is a non-model species, we identified 10 orthologous genes from a well annotated *Brachypodium* genome (a model for C3 grasses), which were also earlier reported for their involvement in drought tolerance. These orthologous candidate genes might play significant roles to help tall fescue persist under drought stress with or without the presence of endophytes.

Candidate gene analysis revealed two orthologous genes, acetylajmalan esterase (*BRADI_4g18187*) and protein altered xyloglucan 4-like (*BRADI_1g44920*), from markers associated with SPD in the EP population. The acetylajmalan esterase is a new member of the Gly-Asp-Ser-Leu (GDSL) lipase/esterase superfamily and plant GDSL lipase/esterase protein family participate in plant responses to biotic and abiotic stresses (Ding et al., 2019). Jaswanthi et al. (2019) investigated drought-induced changes in plant apoplast, which acts as a bridge between environment and plant protoplast and performs multiple functions in metabolism and signaling. LC-MS analysis of apoplast proteome revealed that acetylajmalan esterase was 1.4 fold upregulated under water stress condition in chili (*Capsicum annuum* L.). In *Arabidopsis*, the cuticle destructing factor 1 (*CDEF1*), a member of the GDSL lipase/esterase family of proteins, promote lateral root growth by degrading some components of the cell wall (Takahashi et al., 2009). The endophyte *E. coenophiala* altered root architecture of tall fescue in P deficient soil (Malinowski et al., 1999). Since the endophyte degrades host plant cell wall carbohydrates to gain nutrients as part of their symbiotic relationship (Rasmussen et al., 2012), it might be possible that the endophyte’s activity in cell wall degradation enhance *CDEF1* activity to develop lateral root during stress condition. The protein altered xyloglucan 4-like gene is an orthologs of an *Arabidopsis* gene *AT3G28150*, a member of plant-specific Trichome Birefringence-Like domain and a domain of unknown function 231 (DUF231). Mutation of this gene reduced saccharification yields of biomass and does not impact the plant’s fitness in its natural environment (Gille et al., 2011). The novel endophyte might interact with protein altered xyloglucan 4-like gene to continue their symbiotic relationship with tall fescue.

The dynamin-related protein 4C (*BRADI_1g67517*) was identified from a MTA, which contributed for both SPD and PHT. The dynamin-related protein 4C (DRP4C) is a member of a large multidomain GTPase that regulates membrane fission, fusion, and tabulation during diverse cellular activities, including biogenesis of peroxisomes (Fujimoto and Tsutsumi, 2014). It has been reported that heat and drought increases peroxisome abundance in wheat (Sanad et al., 2019) and quinoa (Hinojosa et al., 2019). Although the molecular function of DRP4C is still unclear, we found the respective marker of this gene controlling 12% phenotypic variation together for PHT and SPD under drought conditions.

The SAC3 family protein C gene (*BRADI_1g62340*) was found from the marker associated with VGR in EP population only. The SAC3 family protein C, a sucrose non-fermenting-1 (*Snf1*)-like serine/threonine kinase gene (Davies et al., 1999), is activated by plant hormone abscisic acid (ABA) in response to drought, saline, and nutritional stress tolerance in *Arabidopsis* and rice (Coello et al., 2010) in order to regulate carbon metabolism. Plants upregulate ABA biosynthesis under drought and heat stresses to reduce the stomatal openings and minimize transpiration. In rice, the symbiotic fungal endophyte *Paecilomyces formosus* LWL1 produces ABA and jasmonic acid that enhance *japonica* rice plant growth under heat stress (Waqas et al., 2015). In this study, identification of SAC3 family protein C gene from V.ep_49 suggests that the presence of endophytes might increase the ABA synthesis of the host grass species under drought stress. The aspartyl protease AED3 (*BRADI_1g19070*), an orthologs of *Arabidopsis AtAED3*, is a cell wall protein expressed in growing organs, such as young leaves and apical internodes of *Brachypodium*, rice, and *Arabidopsis* (Douche et al., 2013). As a cell wall protein, the *AED3* may have a role in abiotic stress response in maize by modifying cell wall structure and being involved in cell wall integrity signaling (Niu and Wang, 2020).

Four orthologous genes were identified in the EF population under natural drought conditions. Among them, the uncharacterized (GPI)-anchored protein (*BRADI_1g42478*) was found in the SV.ef_32 contributing to both SPD and VGR under stress condition. The maize *rth3* (*roothairless3*) encodes a putative GPI-anchor COBRA-like protein that is required for root hair elongation and normal grain yield (Hochholdinger et al., 2008). The GPI-anchor proteins are components of the cell wall and are necessary for cellular integrity, suggesting that GPI-anchor proteins play key roles in root hair formation during drought stress by cell wall biosynthesis. Unlikely, GPI-anchored protein also responded to cold acclimation in *Arabidopsis* (Takahashi et al., 2016). Under drought stress condition, a putative RING-H2 finger protein ALT12 (*BRADI_2g14170*) identified in the S.ef_61 contribute to SPD. Overexpression of the putative RING-H2 finger protein gene, an ortholog in rice designated as *OsRHP1*, enhances drought and salinity tolerance in rice (Zeng et al., 2014). The Phospholipase D (PLD) zeta 1 (*BRADI_2g11807*) gene has been identified from the V.ef_56 contributed to VGR. In rice, the PLD zeta 1 designated as *OsPLD*ζ*1* found to be significantly upregulated under both salt and drought stress conditions (Singh et al., 2012). The candidate gene responsible for DWT identified in the EF population but not found in EP population was Calmodulin-like proteins 5 upregulated in stem tissue under salt and drought stress in *Solanum pennellii* (Shi and Du, 2020). The Calmodulin-like proteins are important Ca^2+^ sensors, which play significant roles in mediating plant abiotic stress tolerance including cold, drought, and salinity (Munir et al., 2016).

In summary, evaluation of clonal pairs of endophyte-positive and -negative tall fescue populations in natural drought stress conditions elucidated that the presence of novel endophyte, AR584, causes reasonable morphological and physiological changes across growing seasons. In this study, a set of 3,597 high quality SNPs was developed in a tall fescue population. GWAS analysis identified 147 putative MTA, of which 26 were specific to drought stress conditions. Among the drought-responsive MTA, 17 were specific to EP, eight in EF, and one was common in EP and EF populations. Candidate orthologous genes (10) in the marker regions were identified using the *Brachypodium* genome. The MTA-derived candidate genes showed enhanced drought tolerance in different crops with or without the presence of an endophyte. However, further research is needed to validate the functions of the identified candidate genes in tall fescue. Identified MTA and candidate genes can be valuable resources to the tall fescue research community.

## Data Availability Statement

The original contributions presented in the study are publicly available. This data can be found here: National Center for Biotechnology Information (NCBI) BioProject database under accession number PRJNA744923.

## Author Contributions

ST and MS conceived and designed the study and critically revised the manuscript. ST organized and implemented the study and conducted the preliminary data analysis. MSI analyzed the data and prepared the manuscript. NK validated the SNPs into *Brachypodium* genome. JC called the initial SNPs from the GBS data. All authors read and approved the manuscript.

## Conflict of Interest

The authors declare that the research was conducted in the absence of any commercial or financial relationships that could be construed as a potential conflict of interest.

## Publisher’s Note

All claims expressed in this article are solely those of the authors and do not necessarily represent those of their affiliated organizations, or those of the publisher, the editors and the reviewers. Any product that may be evaluated in this article, or claim that may be made by its manufacturer, is not guaranteed or endorsed by the publisher.

## Footnotes

1
https://stateimpact.npr.org/texas/drought/

2
http://water.okstate.edu/news-events/news/acs/

3
http://www.sungro.com/professional-product/metro-mix-380/

4
http://www.mesonet.org/index.php/weather/local/

5
https://www.maizegenetics.net/tassel

6
https://www.ncbi.nlm.nih.gov/gene/?term=Brachypodium+distachyon

7
https://www.ncbi.nlm.nih.gov/genome/?term=txid15368[orgn]

## Supplementary Material

Supplementary Figure 1 Histogram of **(A)** plant height, **(B)** plant spread, **(C)** plant vigor, and **(D)** dry biomass weight in NFTD07 tall fescue population.

Supplementary Figure 2 Distribution of 3,597 tall fescue SNPs used for the genome-wide association analysis on *Brachypodium* chromosomes. X-axis indicates chromosome length in Mb, and Y-axis denotes number of SNPs/Mb genomic region. Arrows indicate the centromere position on each chromosome.

Supplementary Figure 3 Principal component (PC) analysis of 205 tall fescue genotypes. The results of the PCs obtained from 3,597 SNP markers.

Supplementary Figure 4 Kinship matrix of the tall fescue panel consist of 205 genotypes.
