# Supplementary material for: Drought Responsive Putative Marker-Trait Association in Tall Fescue as Influenced by the Presence of a Novel Endophyte
Source: Front Plant Sci. 2021 Oct 20;12:729797. doi: 10.3389/fpls.2021.729797 (PMC8565914; doi:10.3389/fpls.2021.729797)

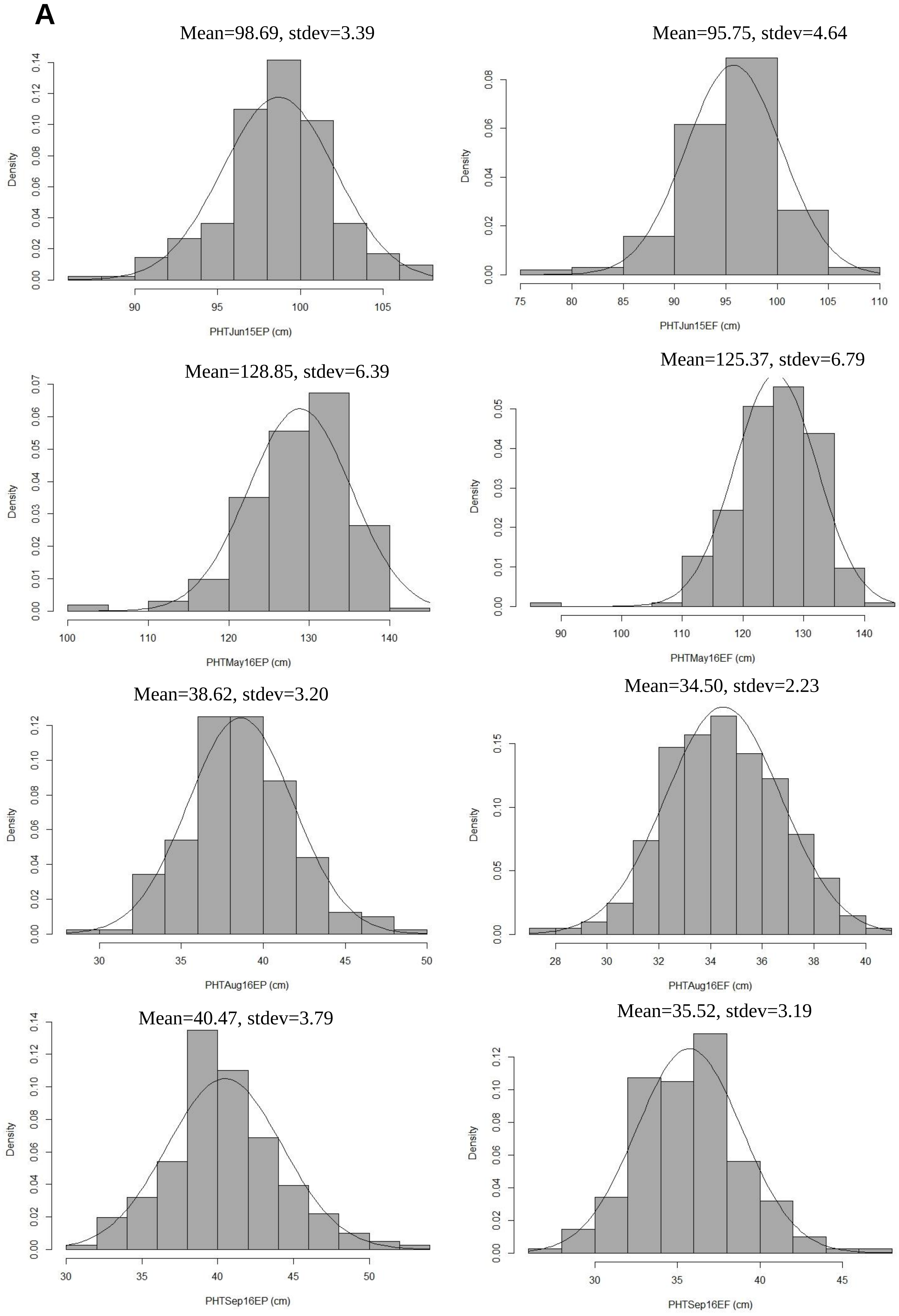
A
Mean=98.69, stdev=3.39
PHTJun15EP (cm)
Mean=95.75, stdev=4.64
PHTJun15EF (cm)
Mean=128.85, stdev=6.39
PHTMay16EP (cm)
Mean=125.37, stdev=6.79
PHTMay16EF (cm)
Mean=38.62, stdev=3.20
PHTAug16EP (cm)
Mean=34.50, stdev=2.23
PHTAug16EF (cm)
Mean=40.47, stdev=3.79
PHTSep16EP (cm)
Mean=35.52, stdev=3.19
PHTSep16EF (cm)
Density

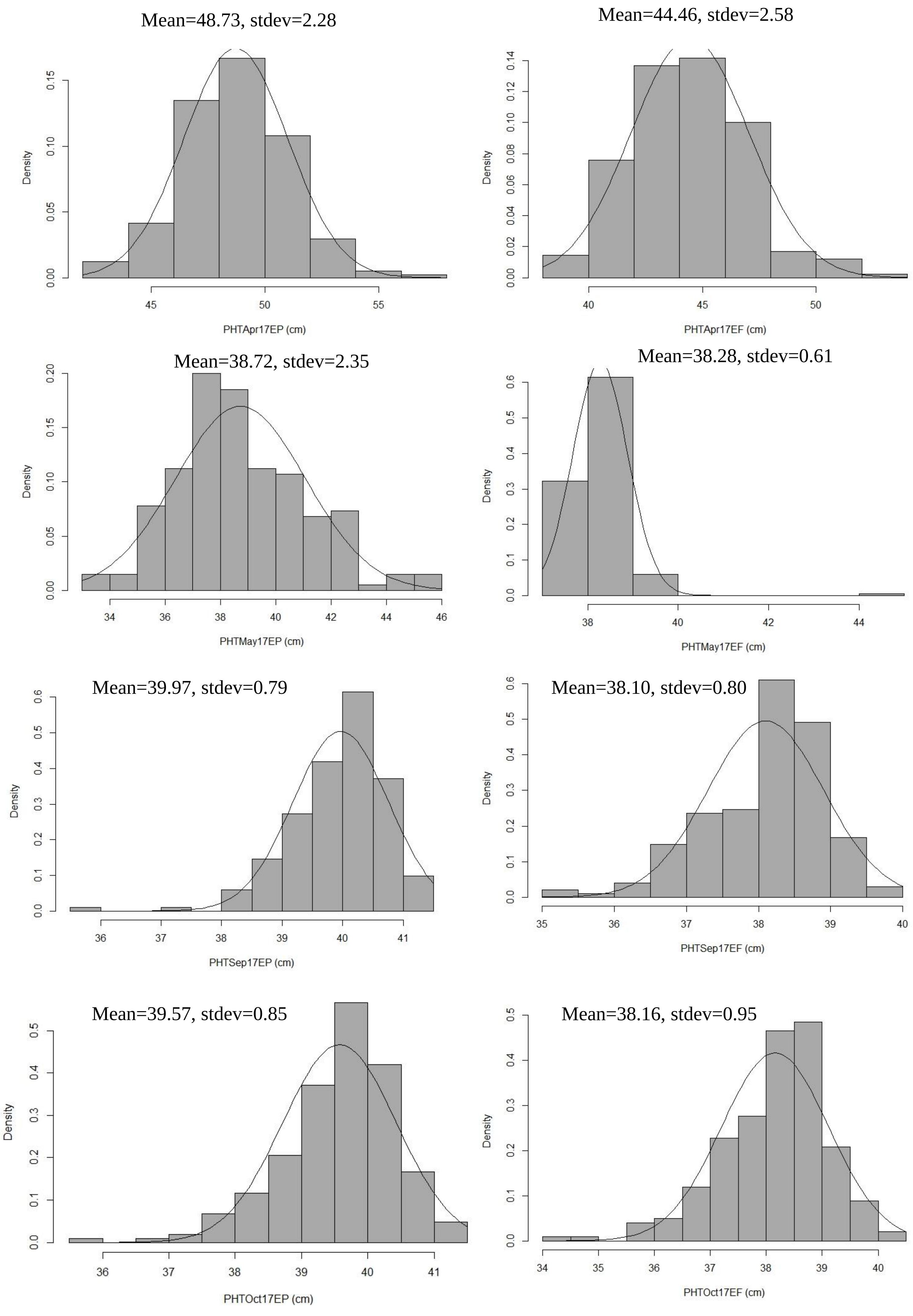
Mean=48.73, stdev=2.28
Density
PHTApr17EP (cm)
Mean=44.46, stdev=2.58
Density
PHTApr17EF (cm)
Mean=38.72, stdev=2.35
Density
PHTMay17EP (cm)
Mean=38.28, stdev=0.61
Density
PHTMay17EF (cm)
Mean=39.97, stdev=0.79
Density
PHTSep17EP (cm)
Mean=38.10, stdev=0.80
Density
PHTSep17EF (cm)
Mean=39.57, stdev=0.85
Density
PHTOct17EP (cm)
Mean=38.16, stdev=0.95
Density
PHTOct17EF (cm)

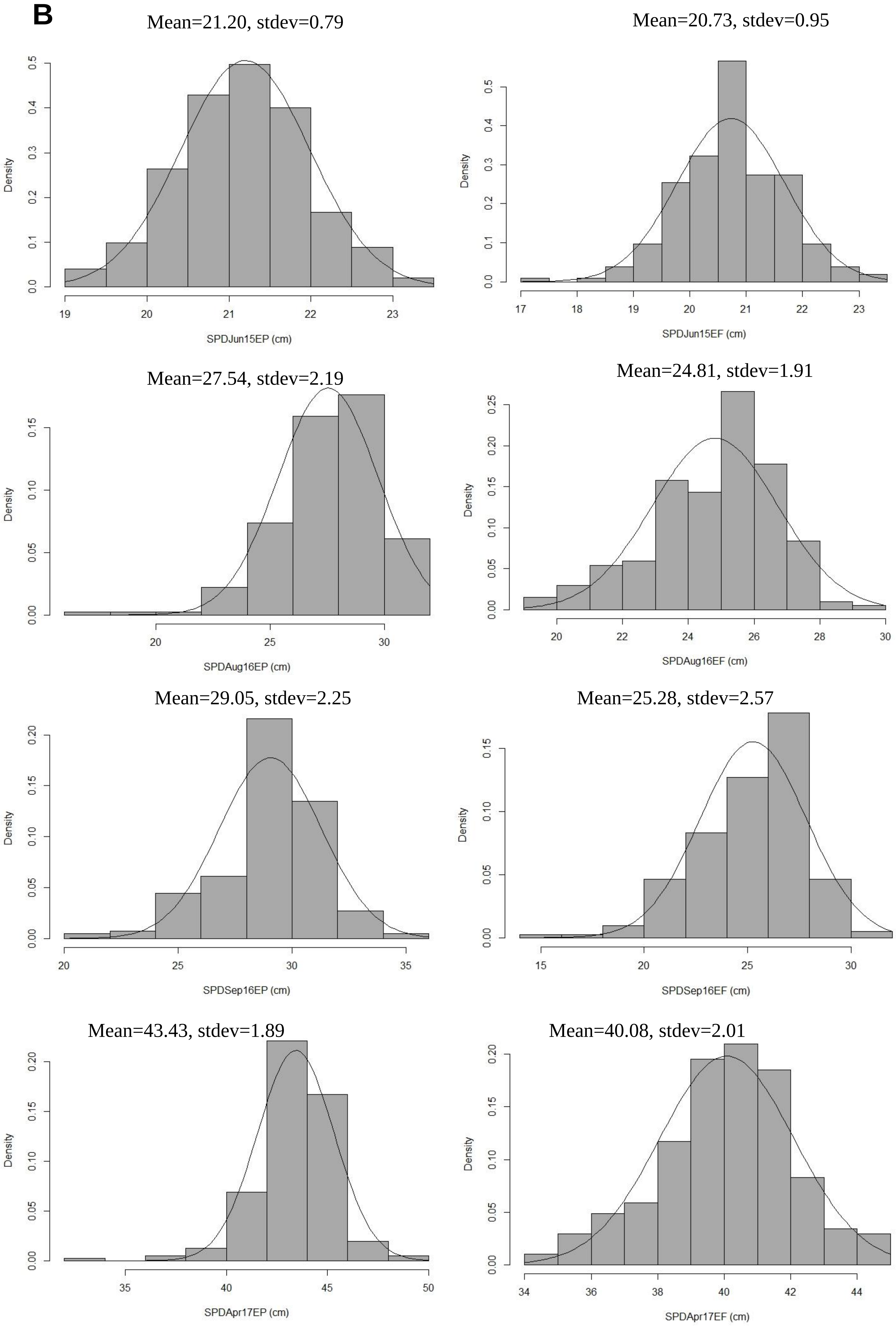
B
Mean=21.20, stdev=0.79
Density
SPDJun15EP (cm)
Mean=20.73, stdev=0.95
Density
SPDJun15EF (cm)
Mean=27.54, stdev=2.19
Density
SPDAug16EP (cm)
Mean=24.81, stdev=1.91
Density
SPDAug16EF (cm)
Mean=29.05, stdev=2.25
Density
SPDSep16EP (cm)
Mean=25.28, stdev=2.57
Density
SPDSep16EF (cm)
Mean=43.43, stdev=1.89
Density
SPDApr17EP (cm)
Mean=40.08, stdev=2.01
Density
SPDApr17EF (cm)

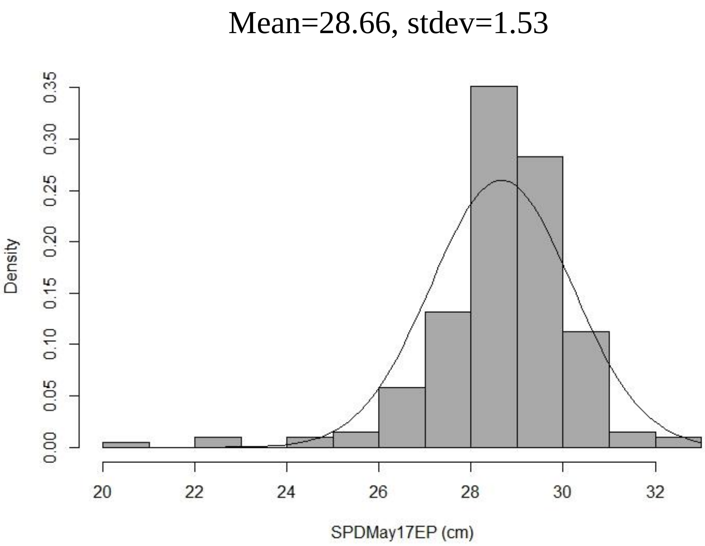
Mean=28.66, stdev=1.53
Density
SPDMay17EP (cm)

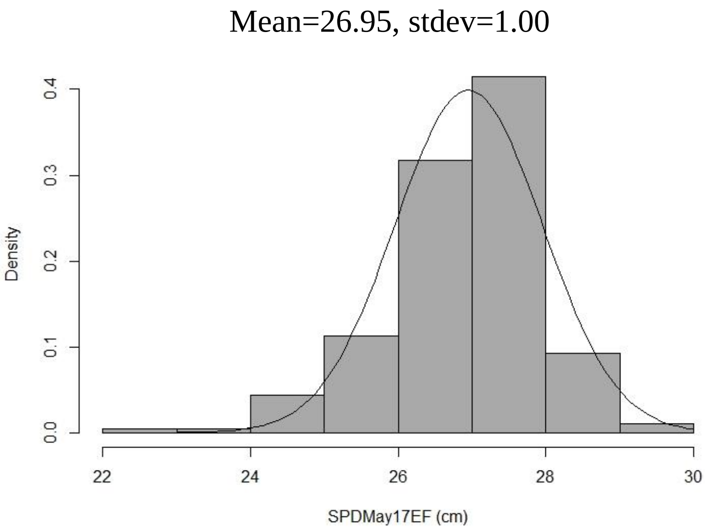
Mean=26.95, stdev=1.00
Density
SPDMay17EF (cm)

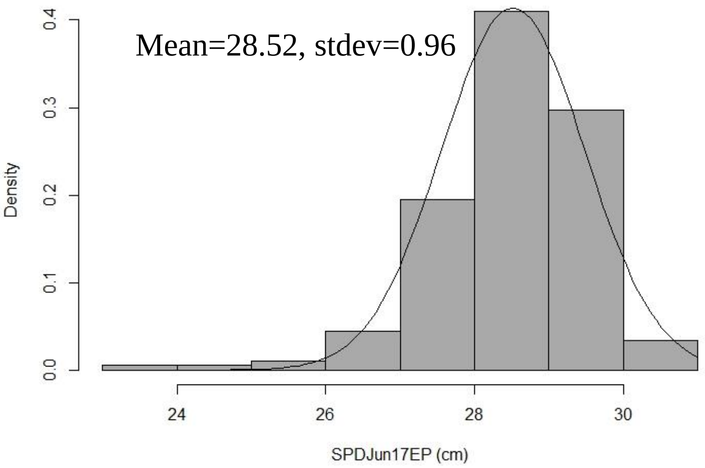
Mean=28.52, stdev=0.96
Density
SPDJun17EP (cm)

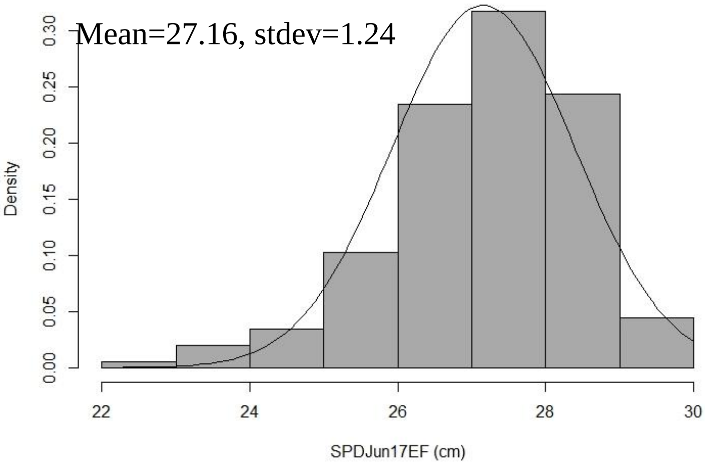
Mean=27.16, stdev=1.24
Density
SPDJun17EF (cm)

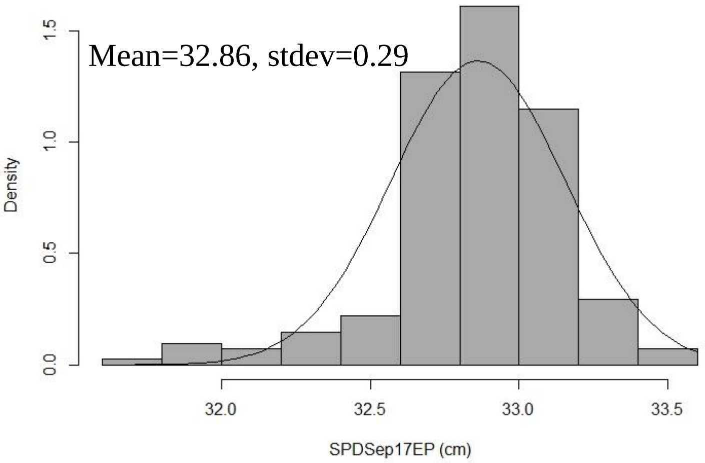
Mean=32.86, stdev=0.29
Density
SPDSep17EP (cm)

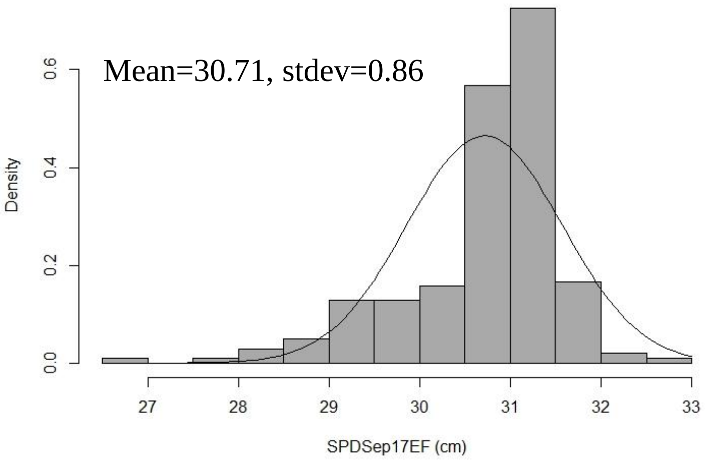
Mean=30.71, stdev=0.86
Density
SPDSep17EF (cm)

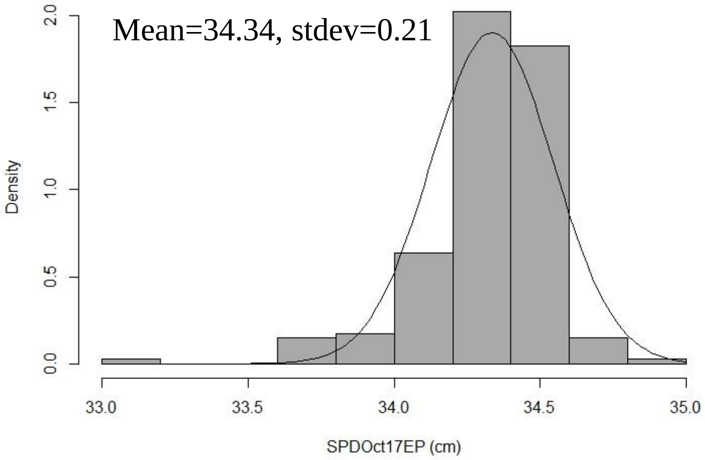
Mean=34.34, stdev=0.21
Density
SPDOct17EP (cm)

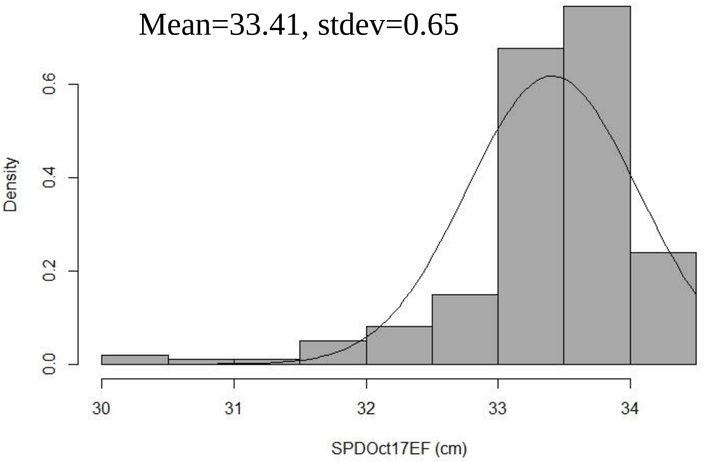
Mean=33.41, stdev=0.65
Density
SPDOct17EF (cm)

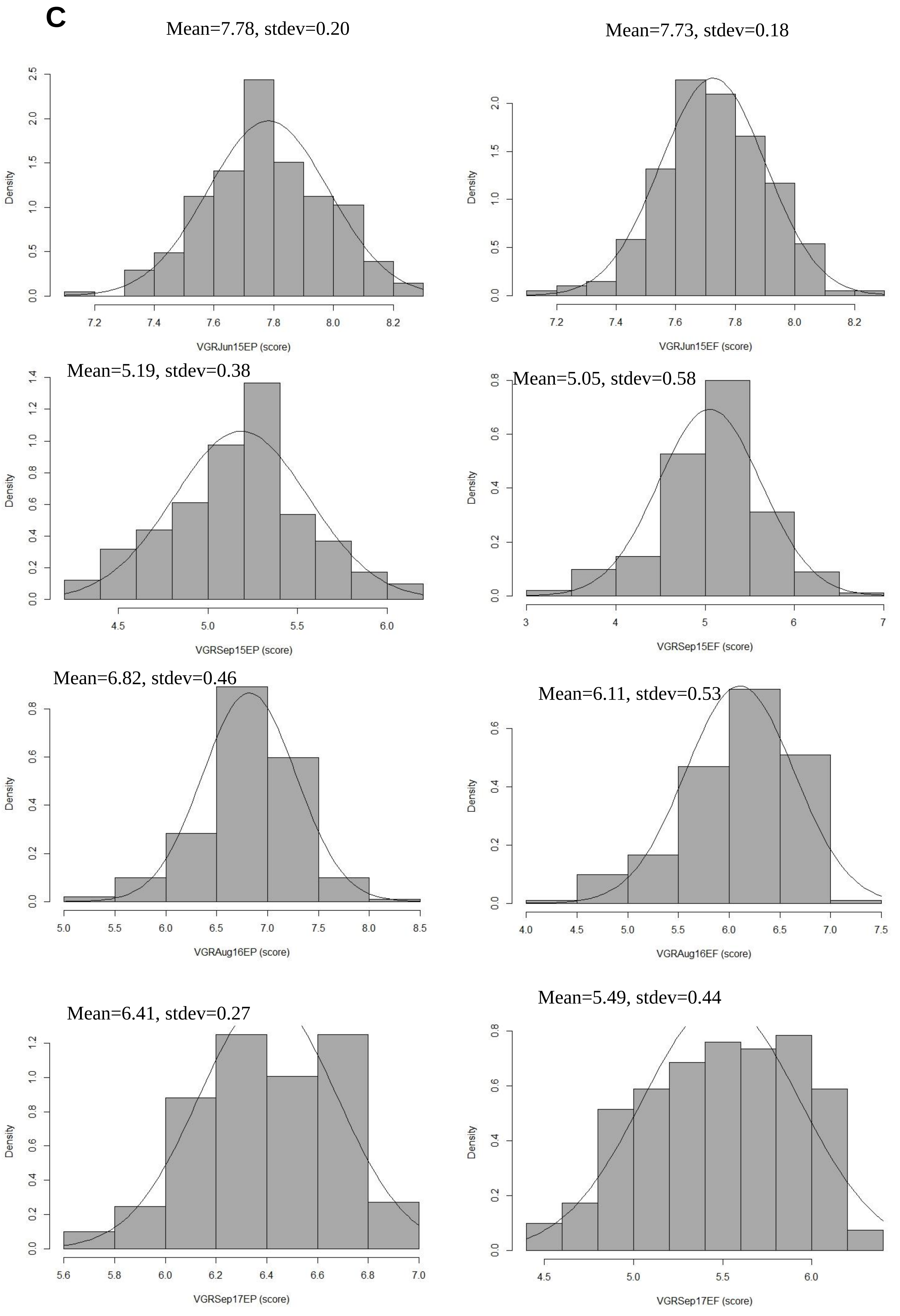
C
Mean=7.78, stdev=0.20
Density
VGRJun15EP (score)
Mean=7.73, stdev=0.18
Density
VGRJun15EF (score)
Mean=5.19, stdev=0.38
Density
VGRSep15EP (score)
Mean=5.05, stdev=0.58
Density
VGRSep15EF (score)
Mean=6.82, stdev=0.46
Density
VGRAug16EP (score)
Mean=6.11, stdev=0.53
Density
VGRAug16EF (score)
Mean=6.41, stdev=0.27
Density
VGRSep17EP (score)
Mean=5.49, stdev=0.44
Density
VGRSep17EF (score)

Mean=6.15, stdev=0.29

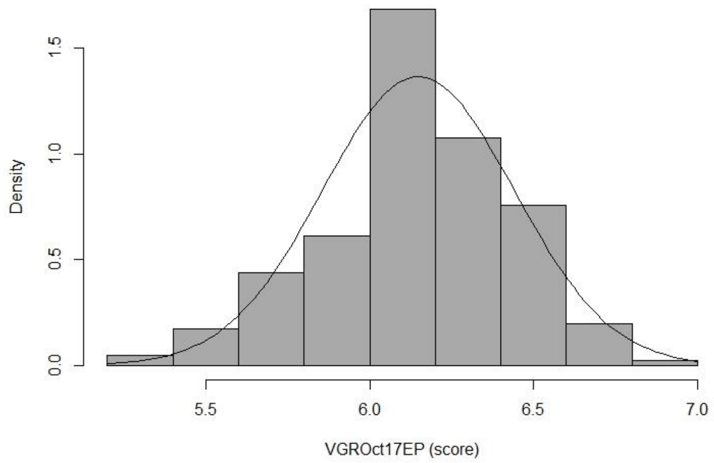

Mean=5.32, stdev=0.44

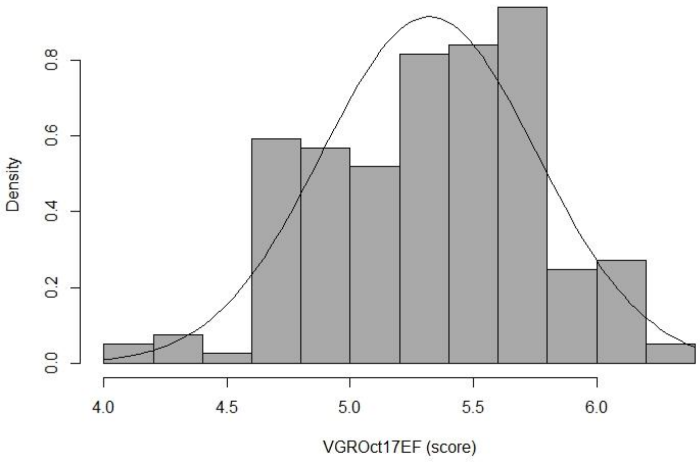

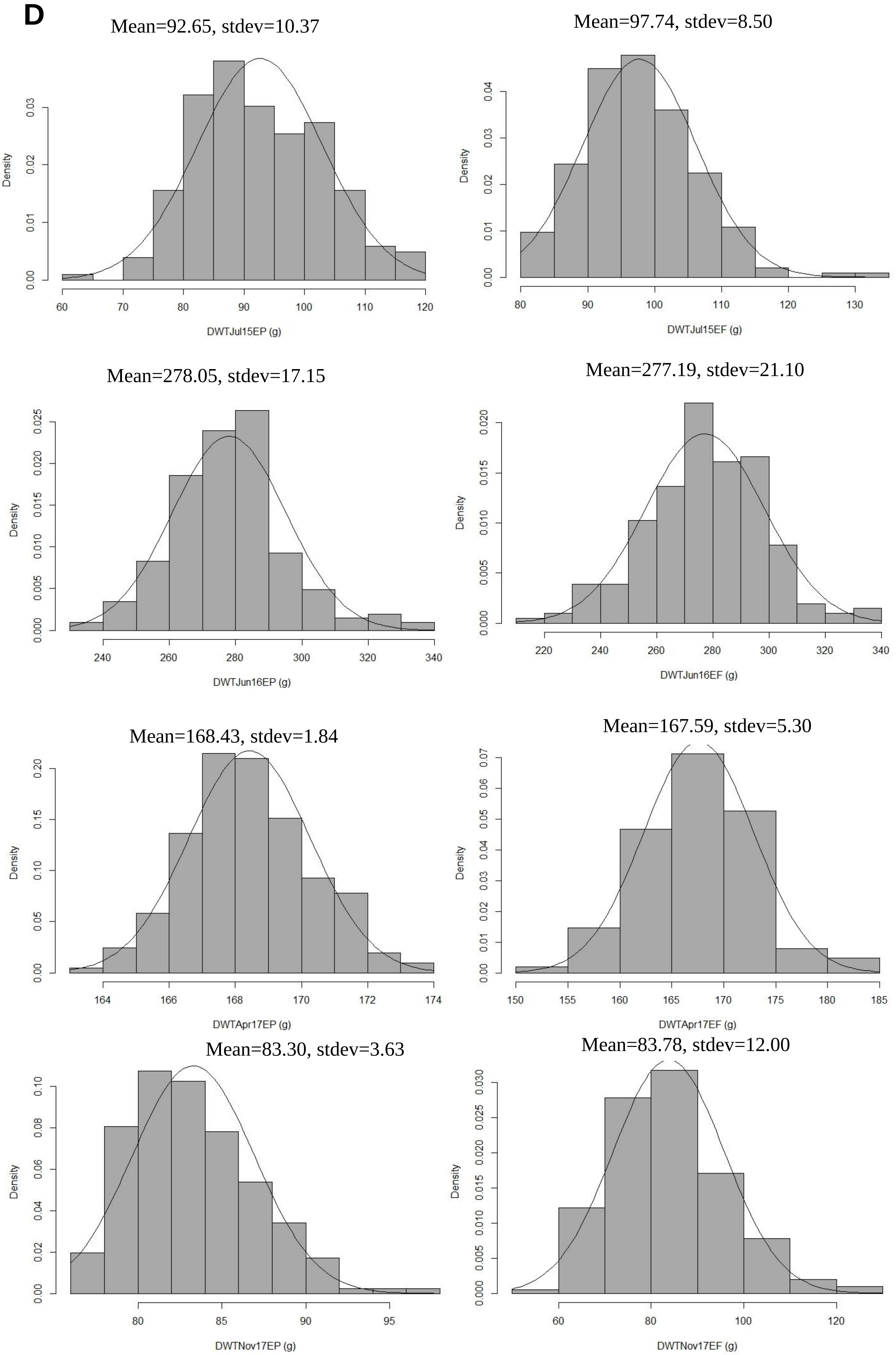
D
Mean=92.65, stdev=10.37
Density
DWTJul15EP (g)
Mean=97.74, stdev=8.50
Density
DWTJul15EF (g)
Mean=278.05, stdev=17.15
Density
DWTJun16EP (g)
Mean=277.19, stdev=21.10
Density
DWTJun16EF (g)
Mean=168.43, stdev=1.84
Density
DWTApr17EP (g)
Mean=167.59, stdev=5.30
Density
DWTApr17EF (g)
Mean=83.30, stdev=3.63
Density
DWTNov17EP (g)
Mean=83.78, stdev=12.00
Density
DWTNov17EF (g)

Mean=77.16, stdev=1.65

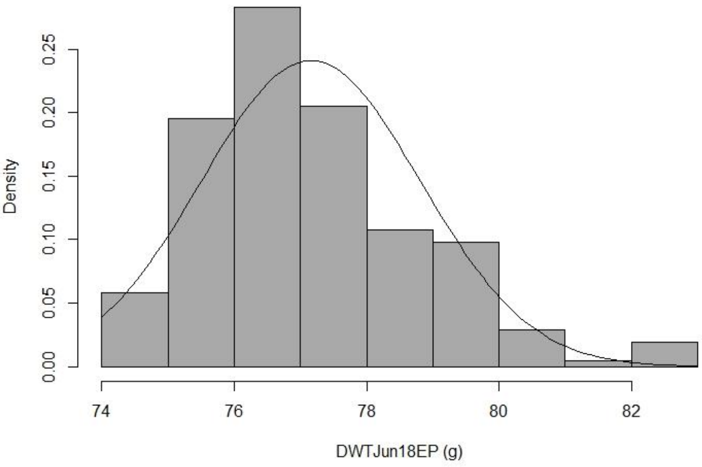

Mean=71.94, stdev=5.83

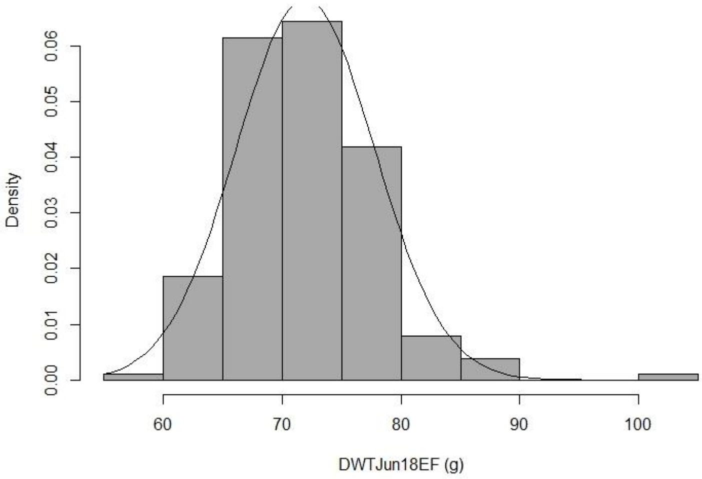

Supplement: Supplementary Figure 1 — Histogram of (A) plant height, (B) plant spread, (C) plant vigor, and (D) dry biomass weight in NFTD07 tall fescue population. [file Data_Sheet_1.zip › Supplementary Figure S1.PDF]
