# Supplementary figures and images for: Drought Responsive Putative Marker-Trait Association in Tall Fescue as Influenced by the Presence of a Novel Endophyte
Source: Front Plant Sci. 2021 Oct 20;12:729797. doi: 10.3389/fpls.2021.729797 (PMC8565914; doi:10.3389/fpls.2021.729797)

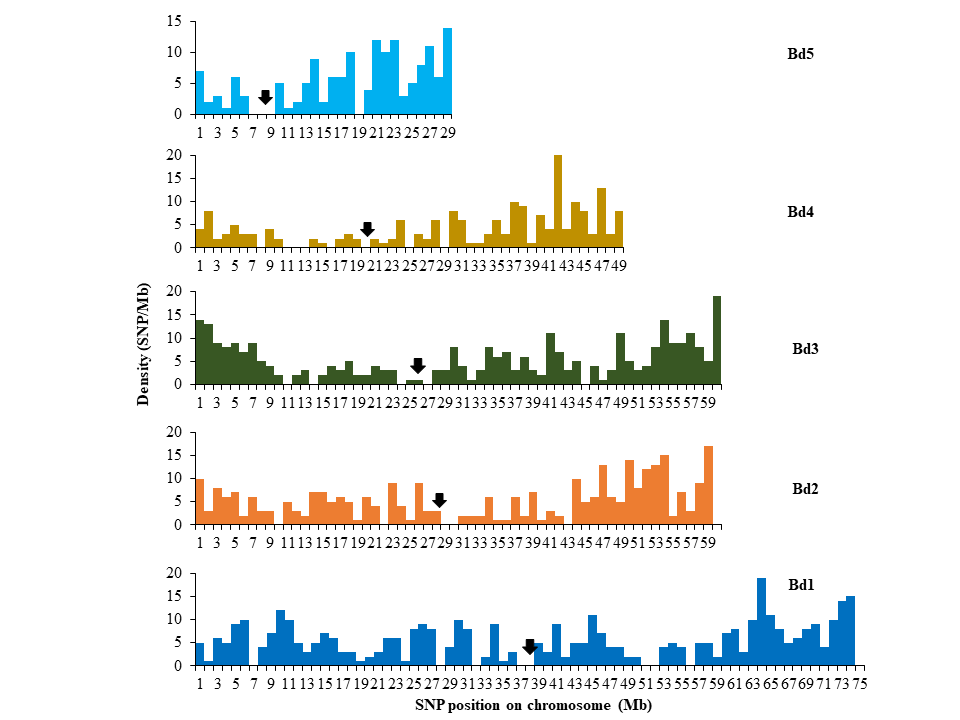

Supplement: Supplementary Figure 1 — Histogram of (A) plant height, (B) plant spread, (C) plant vigor, and (D) dry biomass weight in NFTD07 tall fescue population. [file Data_Sheet_1.zip › Supplementary Figure S2.TIF]

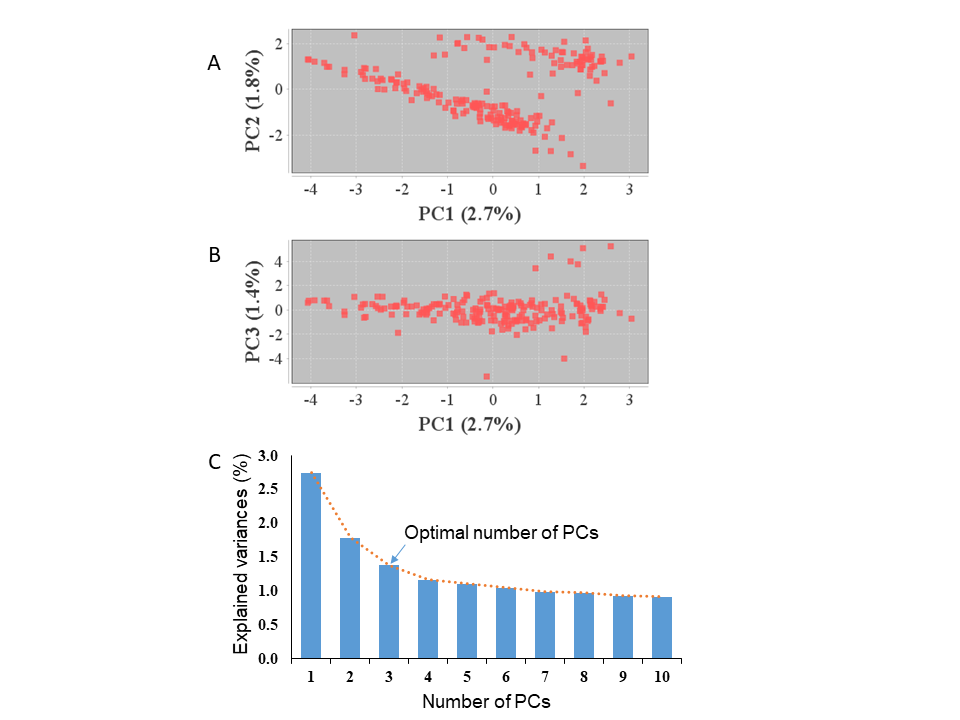

Supplement: Supplementary Figure 1 — Histogram of (A) plant height, (B) plant spread, (C) plant vigor, and (D) dry biomass weight in NFTD07 tall fescue population. [file Data_Sheet_1.zip › Supplementary Figure S3.TIF]

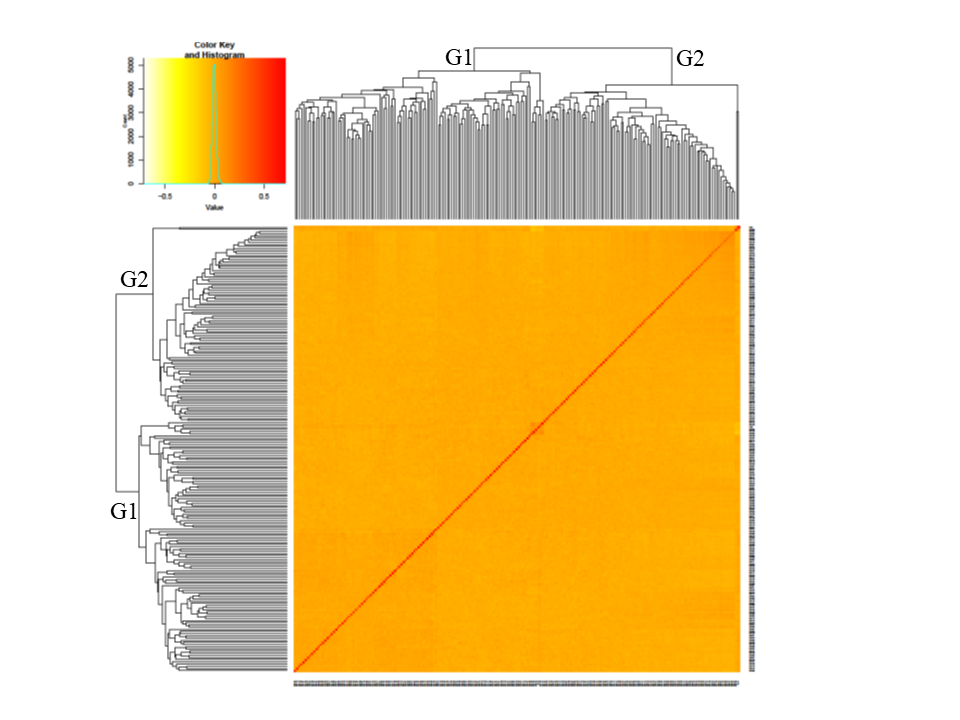

Supplement: Supplementary Figure 1 — Histogram of (A) plant height, (B) plant spread, (C) plant vigor, and (D) dry biomass weight in NFTD07 tall fescue population. [file Data_Sheet_1.zip › Supplementary Figure S4.TIF]
