# Supplementary material for: Drought Responsive Putative Marker-Trait Association in Tall Fescue as Influenced by the Presence of a Novel Endophyte
Source: Front Plant Sci. 2021 Oct 20;12:729797. doi: 10.3389/fpls.2021.729797 (PMC8565914; doi:10.3389/fpls.2021.729797)
Supplement: Supplementary file 2 [file Table_1.DOCX]

**Supplementary Table S1** Weather data of the experimental site from 2014 – 2018 collected from the Mesonet weather station.

|  | Mean monthly air temperature (°C) | | | | | | Total rainfall (cm) | | | | | |
| --- | --- | --- | --- | --- | --- | --- | --- | --- | --- | --- | --- | --- |
|  | 2014 | 2015 | 2016 | 2017 | 2018 | 14-year mean^*^ | 2014 | 2015 | 2016 | 2017 | 2018 | 14-year mean^*^ |
| Jan. | 4.37 | 5.00 | 6.06 | 7.06 | 4.44 | 5.71 | 0.99 | 6.48 | 1.30 | 8.71 | 0.30 | 4.38 |
| Feb. | 4.82 | 4.39 | 11.00 | 12.17 | 6.61 | 7.73 | 2.24 | 2.69 | 3.78 | 5.38 | 18.57 | 4.89 |
| Mar. | 9.97 | 12.89 | 14.17 | 15.44 | 13.89 | 13.40 | 6.53 | 8.99 | 9.22 | 3.45 | 5.99 | 6.93 |
| Apr. | 17.02 | 17.11 | 17.83 | 17.78 | 14.06 | 17.29 | 4.65 | 11.79 | 19.56 | 7.06 | 4.90 | 8.34 |
| May | 21.50 | 19.94 | 20.17 | 21.22 | 24.33 | 21.21 | 4.06 | 55.32 | 17.42 | 8.84 | 17.37 | 16.12 |
| June | 26.15 | 26.33 | 27.39 | 25.39 | 27.5 | 26.86 | 12.42 | 32.05 | 11.00 | 3.99 | 3.68 | 9.87 |
| July | 26.10 | **28.67** | **29.22** | **28.28** | **29.33** | 28.58 | 15.32 | 21.67 | **1.14** | 8.79 | **2.29** | 8.04 |
| Aug. | 26.54 | 27.83 | 27.78 | 25.78 | 27.56 | 28.24 | 3.05 | **0.53** | 10.11 | 16.71 | 10.82 | 6.60 |
| Sep. | 23.02 | 25.39 | 24.67 | 23.67 | 23.39 | 24.06 | 6.15 | 3.68 | 8.00 | **3.25** | 34.44 | 8.27 |
| Oct. | 19.71 | 18.67 | 20.56 | 17.94 | 16.83 | 17.91 | 8.23 | 20.12 | 5.72 | 5.41 | 28.73 | 10.73 |
| Nov. | 8.96 | 12.39 | 13.61 | 13.78 | 9.28 | 12.08 | 11.48 | 19.58 | 5.41 | 0.13 | 1.32 | 5.06 |
| Dec. | 5.54 | 9.39 | 6.22 | 6.5 | 6.78 | 7.29 | 3.63 | 17.83 | 2.16 | 4.90 | 12.78 | 6.01 |
| ^ǂ^Summer  Month’s  mean | 25.45 | 27.06 | 27.27 | 25.78 | 26.95 | 26.93 | 9.23 | 14.48 | 7.56 | 8.19 | 12.81 | 8.19 |

^*^14-year weather data calculated from 2005-2018

^ǂ^Summer month indicates June-September

Bold, highest temperature and lowest rainfall during summer months in each year
